# Early Versus Delayed Androgen Deprivation Therapy for Biochemical Recurrence After Local Curative Treatment in Non-Metastatic Hormone-Sensitive Prostate Cancer: A Systematic Review of the Literature

**DOI:** 10.3390/cancers17020215

**Published:** 2025-01-10

**Authors:** Muneeb Uddin Karim, Steven Tisseverasinghe, Rodrigo Cartes, Constanza Martinez, Boris Bahoric, Tamim Niazi

**Affiliations:** 1 Department of Radiation Oncology, McGill University, Montreal, QC H3A 0G4, Canadaconstanza.martinezramirez@mail.mcgill.ca (C.M.); boris.bahoric.med@ssss.gouv.qc.ca (B.B.); 2 Department of Radiation Oncology, McGill University, Gatineau, QC J8V 3R2, Canada; steven.tisseverasinghe.med@ssss.gouv.qc.ca

**Keywords:** prostate cancer, biochemical recurrence, salvage systemic therapy, treatment timing, prostate-specific antigen failure

## Abstract

Background: The ideal timing of androgen deprivation therapy (ADT) for patients with biochemical recurrence (BCR) of prostate cancer (PCa) remains controversial due to its side effects and uncertain impact on survival outcomes. Methods: We performed a review of the current literature by comprehensively searching the PubMed, Embase, and Cochrane databases to determine the optimal timing of ADT initiation after biochemical recurrence. We selected 26 studies including systematic reviews, randomized controlled trials (RCTs), and retrospective studies, while also reviewing practice guidelines. Results: Not all patients with BCR cancer experience clinical or radiological progression. While early ADT may delay progression, evidence of its effect on PCa-specific mortality remains inconclusive. The PSA thresholds for initiating ADT vary, complicating decision-making. Key predictors of progression include a short PSA doubling time (PSADT), a high Gleason score (GS), and a brief interval to BCR of PCa post-radiotherapy (RT). Combining ADT with androgen receptor pathway inhibitors (ARPIs) has been shown to improve metastasis-free survival in high-risk patients. Conclusion: The ideal timing of ADT initiation in BCR PCa remains uncertain. Early ADT can help control the progression, but its effect on PCa-specific mortality is unclear. Stratifying patients by their risk factors, such as their PSADT, GS, and time to BCR can guide individualized treatment. In high-risk patients, delaying ADT should be avoided, while combining ADT with an androgen receptor pathway inhibitor (ARPI) may further improve outcomes.

## Simple Summary

The ideal timing of androgen deprivation therapy (ADT) for biochemical recurrent (BCR) prostate cancer (PCa) is debated due to its side effects and uncertain survival benefits. Our review of 26 studies found that early ADT may delay PCa progression, but its impact on PCa-specific mortality is still unclear. The PSA thresholds for starting ADT vary, complicating decisions. Key predictors of progression include a short PSA doubling time (PSADT), a high Gleason score (GS), and a brief interval to BCR post-radiotherapy. The combination of ADT with androgen receptor pathway inhibitors (ARPIs) has shown improved metastasis-free survival in high-risk patients. Stratifying patients by their risk factors can guide treatment and, in high-risk patients, delaying ADT should be avoided.

## 1. Introduction

The management of prostate cancer (PCa) following definitive or salvage radiation therapy (RT) hinges significantly on the use of prostate-specific antigen (PSA) monitoring to assess the disease remission. Despite advances in local therapy, biochemical recurrence (BCR) remains a critical clinical challenge. After radical prostatectomy (RP), BCR is defined by two consecutive rising PSA values ≥0.2 ng/mL, while in the case of RT, the Phoenix definition characterizes BCR as a PSA increase of ≥ 2 ng/mL above nadir [1]. BCR rates vary widely: 21–47% at 10 years for RP, 16–52% for external beam radiation therapy (EBRT), and 16–53% at 15 years for brachytherapy [2,3].

**Figure 1:**
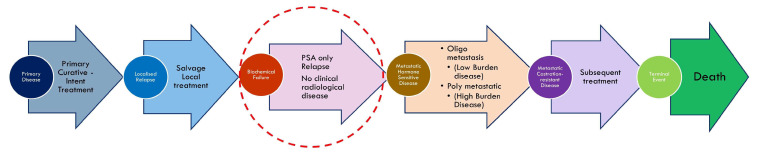
Progression pathways of prostate cancer. PSA—Prostate Specific Antigen.

BCR-PCa represents a state of recurrent disease characterized by rising PSA levels post-local therapy without metastatic disease that is detectable on conventional imaging modalities such as computed tomography (CT), magnetic resonance imaging (MRI), or bone scans. Its natural course is often slow, but progression patterns can differ widely [4] (Figure 1). This variability poses challenges in balancing early therapeutic interventions with possible overtreatment, particularly in asymptomatic patients who may remain free from disease-related complications for years. Importantly, treatment-related adverse effects, including those from androgen deprivation therapy (ADT), can exacerbate comorbid conditions, potentially leading to increased non-cancer-related mortality and a diminished quality of life (QoL) [5,6].

ADT is crucial in suppressing the androgen axis in PCa and is commonly used in patients experiencing BCR after the failure of local therapy. However, ADT can lead to significant adverse effects such as hot flashes, sexual dysfunction, a decreased libido, fatigue, depression, cardiovascular disease, metabolic syndrome, and osteoporosis. Some of these side effects negatively impact patients’ quality of life (QOL), while others can increase mortality by worsening comorbid conditions [5,6]. Although BCR indicates treatment failure, its course can vary among patients, ranging from clinically undetectable, stable, and indolent diseases to more aggressive and progressive patterns that result in poorer outcomes [7,8]. This heterogeneity underscores the complexity of determining the optimal timing for ADT in non-metastatic PSA-only recurrent disease.

The optimal timing of ADT initiation remains a subject of debate. Should ADT commence immediately following PSA recurrence, or should its initiation be deferred until disease progression is evident? Key questions also include identifying the optimal PSA threshold for initiating therapy, understanding significant prognostic factors that influence outcomes, and determining whether deferring ADT could lead to detrimental results [4]. Furthermore, it is essential to assess which patients might benefit from intensified treatment with androgen receptor pathway inhibitors (ARPIs) [9]. Despite its critical role, current practices surrounding ADT initiation are hampered by limited large randomized controlled trials (RCTs) and the lack of consistent evidence supporting an overall survival (OS) benefit in this context. Additionally, ADT-related toxicities and the consequent reduction in QoL necessitate careful consideration. However, emerging data suggest that earlier and more intensified therapy could be advantageous, particularly for patients with high-risk BCR [10,11,12]. Identifying individuals who would benefit most from early ADT initiation and intensified treatment strategies remains a pressing need.

This review seeks to address these gaps by providing a comprehensive analysis of early versus delayed ADT initiation in patients with non-metastatic PCa who are experiencing PSA-only recurrence. It aims to evaluate the clinical effectiveness of different timing strategies, explore prognostic factors that influence outcomes, and assess the evolving role of ARPIs. By linking these insights to practical applications, this study aims to guide clinicians in optimizing treatment timing and improving patient outcomes.

## 2. Materials and Methods

**Figure 2:**
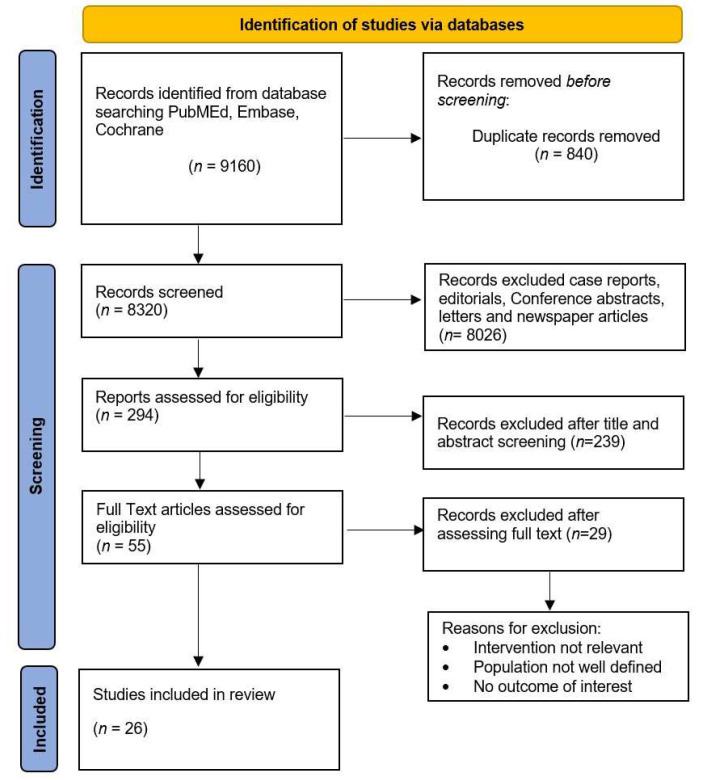
PRISMA flow diagram.

We conducted a systematic review in accordance with PRISMA (Preferred Reporting Items for Systematic Reviews and Meta-Analyses) guidelines, as shown in Figure 2.

The PubMed, Embase, and Cochrane databases were systematically searched for articles from January 2000 to December 2023 using predefined keywords: “prostate neoplasm”, “androgen deprivation”, “androgen receptor antagonists”, “biochemical recurrence”, “salvage therapy”, “time to treatment”, “delayed treatment”, “prostate-specific antigen progression”, and “treatment failure”. The review protocol was registered on Prospero ID# CRD42023416046 Advanced queries in PubMed and Embase were limited to articles written in English or with an available English translation, as shown in Table 1.

This review selectively included studies that addressed critical aspects of BCR PCa management. Additionally, references from selected articles were hand-searched for relevant studies that were included as appropriate.

### 2.1. Inclusion Criteria

This review focused on studies examining the timing of ADT in patients with BCR-PCa who show no clinical or radiological evidence of metastasis. It included studies evaluating the timing of ADT in individuals who had undergone primary local or salvage treatments, such as RP or RT. Additionally, investigations exploring the integration of ARPIs, as well as reports identifying prognostic factors that influence the timing of ADT initiation, were also reviewed.

### 2.2. Exclusion Criteria 

This review excluded phase I/II studies and those that did not address the timing of ADT for BCR-PCa. Studies involving chemotherapy, those focused on metastatic hormone-sensitive or castrate-resistant prostate cancer (PCa), and works addressing pathologies other than prostate adenocarcinoma were also excluded. Additionally, conference abstracts and non-experimental publications, such as narrative reviews, letters to the editor, opinion pieces, and book chapters, were omitted to maintain a focus on evidence-based and experimental research.

### 2.3. Data Extraction

Essential data were systematically extracted from each study included in our review:

1. General Information: key details such as the primary researcher, year of publication, size of the patient cohort, and median follow-up duration were recorded;
2. Methodology: information on the study design, total number of patients, and prior local treatments received was collected to understand the context and scope of each study;
3. Intervention: specific data related to the timing of ADT initiation, the PSA levels at the start of treatment, definitions of early versus delayed ADT, and any relevant prognostic factors were documented;
4. Results: outcomes were evaluated for efficacy, focusing on prostate cancer-specific mortality (PCSM), freedom from distant metastases (FDM), cancer-specific survival (CSS), and overall survival (OS).

Regular consensus meetings were convened among the authors to discuss publications, extract pertinent information from selected manuscripts, and resolve any disagreements encountered during the review process. Investigators confidently assessed the risk of bias independently using clear, predefined criteria. Any disagreements were effectively resolved through consensus, ensuring the integrity of the evaluation. This collaborative approach ensured the accuracy and reliability of the data extracted for analysis.

## 3. Results

### 3.1. Search Results

This systematic review comprehensively evaluated pertinent clinical studies and guidelines relating to the timing of ADT in BCR. Following abstract screening, 55 articles were selected for full-text review. The final inclusion comprised 2 systematic reviews, 3 randomized controlled trials, and 21 retrospective studies. A total of 26 studies, encompassing retrospective analyses, phase III trials, and systematic reviews, were identified. Table 2 provides a succinct overview of the selected studies that investigated the timing of ADT following surgery, with or without adjuvant salvage RT. Table 3 and Table 4 delineate the studies that examined ADT timing post-RT and post-surgery, respectively, while Table 5 presents studies that elucidate the role of ARPI in this context.

### 3.2. Study Analysis

The effectiveness of ADT was assessed in one systematic review and two RCTs. Additionally, the impact of ARPIs was explored in one published RCT. Furthermore, a meta-analysis accompanying a systemic review comprehensively examined prognostic factors linked to BCR. Among the retrospective studies analyzed, 12 investigated outcomes following definitive RT, including one focusing on patients treated with definitive brachytherapy. Moreover, four studies specifically evaluated ADT post-surgery, while five studies included patients undergoing combined surgery with or without RT. Notably, all of these studies adopted a retrospective design.

### 3.3. Definition of Intervention

#### 3.3.1. Definition of Early Treatment

The criteria defining the early treatment of PCa with ADT varied notably among studies. Some studies specified early treatment as commencing ADT within specific timeframes, such as <3 months [16,21], <8 weeks [13], or <12 months [17]. Moreover, the thresholds for PSA levels above nadir that indicated the initiation of early ADT also varied across studies: 2 ng/mL [15,36], 3 ng/mL [14,23], or <10 ng/mL (*n* = 6) [19,20,25,30,31,32] were considered early. Furthermore, one retrospective study also classified a PSA range of 10–20 ng/mL as early [18]. In studies focusing solely on post-surgery cases, PSA levels of 5–10 ng/mL [32] and 0.4 ng/mL [33] were mentioned as thresholds for early treatment. Furthermore, the indication for early ADT treatment based on rapid PSA doubling time (PSADT) ranged between studies from <3 months to <12 months [10,22,24,26,34,35].

#### 3.3.2. Definition of Delayed Treatment

The definition of delayed ADT following PSA recurrence exhibited heterogeneity among studies, with variations such as >3 months [21], >12 months [17], >24 months, or at clinical failure [13,16,23,29,35]. Similarly, the PSA thresholds above nadir that were considered indicative of delayed treatment differed between studies, such as >2 ng/mL [10,15], >10 ng/mL [20,25,30,31,32], and >20 ng/mL [19]. PSADT was also utilized for considering delayed treatment and showed variability across studies, with criteria such as >6 months [24] and >12 months [22].

### 3.4. Outcomes/Endpoints

The studies primarily focused on survival outcomes, with common endpoints including the OS, cause-specific survival (CSS), all-cause mortality (ACM), PCSM, progression-free survival (PFS), freedom from distant metastasis (FFDM), progression to castration-resistant prostate cancer (CRPC), and QOL. The RCTs specifically assessed OS, QoL, time to CRPC, and metastasis-free survival (MFS) outcomes.

### 3.5. Observation of Outcomes

#### 3.5.1. Results from Retrospective Series

Interstudy variability was observed in the time to initiate ADT and in the methodologies used to measure survival outcomes. For instance, Ahn et al. found that delaying ADT until the PSA reached ≥2 ng/mL increased the risk of developing CRPC and mortality [15]. Similarly, Kestin et al. suggested that a PSA elevation of 2 or 3 ng/mL above nadir was optimal for identifying clinically significant BCR and initiating ADT intervention [23]. Klayton et al. confirmed that immediate ADT in patients with a PSADT ≤6 months improved CSS, with less clear benefits in patients with longer PSADTs [24].

In contrast, Garcia-Albeniz et al. found no difference in survival outcomes between immediate and deferred ADT initiation [16]. Sagalovitch et al. reported no benefit of delayed salvage ADT over immediate therapy but noted the importance of the PSADT and time to BCR as prognostic factors [21]. Fu et al. did not observe any impact of ADT on overall survival or PCSM except in men with very rapid disease progression, defined as those with a PSADT <9 months [17]. Kim-Sing et al. suggested that a rapid PSADT was a marker of poor prognosis in patients with BCR after EBRT [26].

Martin et al. proposed observation as a viable option for men older than 76 years with significant comorbidities who experience BCR after RT and have a PSADT > 2 years [28]. Tenenholz et al. argued that ADT could improve survival in patients with a PSADT around ≤7 months [29]. Souhami et al. reported that early salvage ADT improved OS but not cancer-specific mortality or local failure [30]. Mydin et al. showed that early salvage ADT based on a PSA ≤10 ng/mL and no distant metastases increased the survival in patients with PCa who failed neoadjuvant ADT with RT [31]. Moul et al. found that early ADT was beneficial only for high-risk patients experiencing BCR after RP, while Antonarakis et al. and Marshall et al. reported that delaying ADT until metastasis did not compromise survival outcomes [32,34].

#### 3.5.2. Results from Systematic Reviews

The European Association of Urology (EAU) conducted a systematic review to evaluate the effectiveness of salvage ADT in patients with recurrent non-metastatic BCR, focusing on those deemed ineligible for local salvage treatment [7]. The review encompassed 27 studies with 11,606 patients and primarily assessed the OS over 5 years and until last follow-up. The secondary outcomes included CSS, FFDM, symptom-free survival, time to second-line systemic therapy, associated adverse events, QOL, and pain. Several prognostic factors were identified, including a short PSADT, a high GS, high PSA levels, an increased age, and the presence of comorbidities.

Another systematic review and meta-analysis aimed to identify prognostic factors by examining the survival in patients with BCR. Factors such as the age, the PSADT, the interval to BCR, positive surgical margins, the T-stage, the initial PSA, the GS, the salvage ADT, and the salvage RT were assessed [8]. A short PSADT was identified as the main prognostic factor for distant metastatic recurrence. The authors proposed a classification system based on these factors for prognostication, regrouping patients into low- and high-risk categories based on certain clinical risk factors.

#### 3.5.3. Results from RCTs

The TROG 03.06 trial, a phase III RCT, investigated the optimal timing for initiating ADT in patients with PCa with PSA-only progression [13]. The trial comprised two patient groups: one with BCR after RT or RP with or without adjuvant RT, and another consisting of asymptomatic patients deemed unsuitable for curative treatment at diagnosis and who had not received prior ADT. In the immediate treatment arm, ADT commenced within 8 weeks of randomization, while in the delayed treatment arm, it was initiated 2 years later, unless patients developed symptoms, metastases, or had a PSADT of ≤6 months. A total of 293 patients were randomized, with 261 in the first group and 32 in the second. Among them, 151 patients received delayed ADT (137 in the first group and 14 in the second), while 142 patients received immediate ADT (124 in the first group and 18 in the second). At a median follow-up of 5 years, the OS was significantly higher in the immediate ADT group compared to the delayed group, with rates of 91.2% (95% confidence interval [CI] 84.2–95.2) and 86.4% (95% CI 78.5–91.5), respectively (log-rank *p p* = 0.047). The most common serious adverse events were cardiovascular events, reported in 6% of the delayed group and 9% of the immediate group. However, there were no significant differences in grade 3 toxicity or treatment-unrelated adverse events requiring hospital admission between the two groups. Although both groups experienced a decrease in quality of life, the differences were not statistically significant. The authors concluded that immediate ADT provided a small but significant survival benefit in the initial years for patients with BCR after curative treatment or those with non-curable PCa, with only a minor deleterious impact on quality of life. Meanwhile, Crook et al. conducted an RCT to compare continuous and intermittent hormonal therapy (HT) in patients with BCR after primary therapy [14]. After a median follow-up of 6.9 years, the study found no significant difference in OS between the two HT strategies (median OS: 8.8 years vs. 9.1 years; *p* = 0.009 for non-inferiority; HR: 1.02 [0.86–1.21]), irrespective of the GS. The PSA recurrence threshold in the study was >3.0 ng/mL.

#### 3.5.4. Combined Therapy with ARPI and ADT

The recently published EMBARK RCT demonstrated that adding enzalutamide to ADT improved the MFS in men with high-risk BCR compared to ADT alone [10]. The trial enrolled 1068 men who met high-risk criteria, including a PSADT ≤9 months and a PSA ≥ 2 ng/mL after RT or ≥1 ng/mL after RP. Enzalutamide and ADT were discontinued at week 37 if the PSA was ≤0.2 ng/mL, and resumed if the PSA reached ≥5 ng/mL without RP or ≥2 ng/mL with RP. After five years, the combination group had a higher MFS rate than the ADT-alone group (87.3% vs. 71.4%). Additionally, the combination group exhibited a better OS rate than the ADT-alone group (92% versus 87%; HR 0.59, 95% CI 0.38–0.91). However, more patients discontinued treatment due to adverse events in the combination group (21%) and enzalutamide-alone group (18%) than patients who discontinued treatment due to adverse events in the leuprolide-alone group (10%). Common toxicities included hot flashes, fatigue, and arthralgias, with gynecomastia occurring much more frequently in the enzalutamide-alone group (45% vs. 8–9%). While enzalutamide alone reduced the risk of metastasis by 9% when compared to leuprolide alone (71% vs. 80%), it did not improve the OS. However, the combination of enzalutamide and leuprolide improved both the MFS and OS compared to leuprolide alone, suggesting a potential benefit of combined therapy in high-risk BCR. 

## 4. Discussion

The optimal timing and impact of early versus delayed ADT after BCR following local/salvage RT remains a controversial topic. Previous studies attempting to address this question have failed to provide clear guidelines. However, many studies have revealed that the prognosis of BCR often depends on individual patient characteristics, as well as clinically important prognostic variables such as a short PSADT, a high GS, high PSA levels, and the time to BCR. Hence, the entire clinical picture must be assessed prior to initiating early ADT in patients with PSA-only recurrence. Indeed, for patients with a baseline PSA level between 8 and 50 ng/mL, the risk of death from PCa was approximately 7.5 times higher in patients with a PSADT ≤12 months compared to patients with a PSADT of ≥12 months [37]. In a retrospective analysis of patients with BCR post-RP, the authors concluded that stratifying patients into high- and low-risk groups for PCSM could be achieved by considering variables such as the pathological GS, time from surgery to BCR, and PSA-DT. In this study, patients with a PSADT of ≥3 months had a median survival of 6 years. However, patients with a PSADT of ≥3 months who also had BCR occurring within 3 years after surgery and a pathological GS of 8–10 had a median survival of only 3 years. Conversely, patients with a PSADT of ≥15 months and BCR occurring ≥3 years after surgery achieved 100% cause-specific survival [36]. 

While several studies have examined the natural history of BCR following RP, only a limited number of studies have investigated the natural progression of BCR following RT. In a retrospective study of 2,694 men who underwent EBRT for localized PCa, 609 men (22.6%) experienced BCR and the cumulative incidence of PCSM after BCR at 5 years was found to be only 18%. Factors such as a shorter time to BCR (<3 years), shorter PSADT (<3 months), higher initial clinical stage (cT3b/4), and higher pre-treatment GS were significant predictors of clinical progression, DM, and PCSM [38]. Hence, the occurrence of late deleterious patient outcomes post-BCR such as clinically significant progression or mortality can correlate with these risk factors.

The systematic review conducted by the European Association of Urology (EAU) as part of its 2016 guidelines revealed conflicting results regarding the effectiveness of ADT in this setting. The authors thoroughly reviewed studies involving heterogeneous populations and outcomes (post-RP, post-RP +/− adjuvant RT, or post-RT) to determine the clinical effectiveness of early ADT and identify associated prognostic markers [7]. Two RCTs included in this review were the TOAD and PR7 trials, which primarily focused on comparing intermittent versus continuous ADT approaches in this setting. The review indicated that only a minority of patients with BCR will experience systemic progression. Therefore, not all patients with BCR should be immediately initiated on early ADT, considering the adverse side effects and negative impact on QOL associated with ADT. However, predictive factors such as a short PSADT, a high GS, elevated PSA levels, an advanced age, and comorbidities were found to be significant and should be taken into consideration when making treatment decisions given their substantial influence on outcomes related to PCa, including the CRPC, DM, CSS, and OS.

The TOAD study, a large phase III RCT, sought specifically to determine the optimal timing of ADT after BCR [13]. In this study, 63.2% of patients had BCR post-RT while 36.8% had BCR post-RP with or without postoperative RT. The 5-year OS rates were 91% for the early ADT group and 86% for the delayed ADT group, with a hazard ratio (HR) of 0.55 (95% CI, 0.3 to 1; *p* = 0.05) for the total cohort. When considering the subgroup of patients with PSA-only relapses, both groups showed good 5-year OS rates (78.2% for delayed therapy and 84.3% for immediate therapy), with an HR of 0.58 (95% CI, 0.3 to 1.12; *p* = 0.1). Importantly, 41% of men in the delayed ADT group did not require further therapy, while those in the immediate ADT group experienced increased short-term toxicity. Furthermore, no difference was observed in the PCSM between the immediate and delayed ADT groups. However, men receiving immediate ADT had a longer time to local and distant progression. The trial initially planned to enroll 750 patients and required 353 events. Unfortunately, due to poor enrollment, the trial was prematurely closed, and only 46 deaths occurred during the study. Consequently, the trial was underpowered, making it difficult to draw definitive conclusions. Nonetheless, the trial suggests that immediate ADT may delay local and distant progression without indicating clear benefits for OS or PCSM in patients with non-metastatic BCR.

The ELAAT trial (NCT00439751) had a similar design, but also fell short of reaching its accrual goal. The authors of both the TOAD and ELAAT trials presented a combined analysis of these two randomized phase III trials in an abstract at the ASCO 2018 meeting. A total of 261 patients from the TOAD trial and 78 patients from the ELAAT trial were followed for a median duration of 5 years and there were no statistically significant differences in the ACM (HR 0.75, 95% CI 0.40, 1.41; *p* = 0.37) or CSM (HR 0.57, 95% CI 0.22–1.49) between the two treatment groups [39]. 

For a long time, ADT alone has been the only available treatment option in this setting. With potent ARPIs, new strategies are emerging. The EMBARK study showed that adding enzalutamide, a nonsteroidal anti-androgen to ADT, improved outcomes for high-risk BCR patients. This has led to a shift in the treatment approach for this patient population. The data from this trial confirm the advantage of ARPIs as early as non-metastatic HSPCa (hormone sensitive prostate cancer). Indeed, at five years, the MFS rate was 87.3% with ADT + enzalutamide, 71.4% with ADT alone, and 80.0% with enzalutamide monotherapy. Thus, EMBARK indicates that combined therapy (HR 0.42; *p* < 0.001) and enzalutamide monotherapy (HR 0.63; *p* = 0.005) are superior to ADT alone in this setting. Patient-reported outcomes indicated that combined therapy and enzalutamide monotherapy, when compared to ADT alone, preserved a high health-related QoL. The response-related treatment pauses ranged from 11 months with enzalutamide to 20.2 months with enzalutamide and ADT for patients with PSA decreases <0.2 ng/mL at week 37, demonstrating a more durable response in the combined group. Notably, treatment was suspended at week 37 in 321 (91%) patients in the enzalutamide combination arm and 240 (68%) patients in the leuprolide alone-arm, based on their PSA response [10]. Thus, this study establishes ARPIs as the new standard of care for patients with high-risk BCR.

In the PRESTO phase III RCT, the authors investigated the impact of different ARPIs +/− ADT in high-risk BCR patients with PCa. The trial recruited 504 patients that were allocated to three groups: ADT alone, ADT + apalutamide (APA), or ADT + APA + abiraterone acetate plus prednisone (AAP). The main outcome was biochemical PFS (bPFS). Secondary outcomes included safety, QOL, time to testosterone recovery, MFS, and time to castration resistance. The interim analysis reported that both experimental groups (ADT + APA and ADT + APA + AAP) had longer bPFS than the control group (median bPFS of 24.9 mo for ADT + APA vs. 20.3 mo for ADT; HR = 0.52 (95% CI: 0.35–0.77); median bPFS 26.0 months for ADT + APA + AAP vs. 20.0 mo for ADT; HR = 0.48 (95% CI: 0.32–0.71), with acceptable safety and no effect on testosterone recovery. However, while these results are promising, improvement in bPFS is less impactful than improvements in MFS or OS. The initial report indicates that a more complete androgen receptor blockade with APA and ADT can improve outcomes for high-risk BCR patients, and that stronger ADT should be considered in this setting. The MFS and OS have not yet reached maturity and are expected to be reported in the future [11]. 

Currently, there is no consensus on the exact PSA threshold at which early ADT should be initiated. A survey of Canadian uro-oncologists evaluating their practice patterns for patients with PSA-only BCR post-RT showed that approximately half of the respondents start ADT when the PSA level exceeds 10 ng/mL or when the PSADT is less than 6 months [40]. However, the study by Mahal et al. [20] demonstrated that initiating ADT at a later stage, with a PSA ≥ 12 ng/mL, was associated with an increased risk of PCSM. Only three studies, included in our systematic review, provided data on the PSA threshold for initiating early ADT (PSA ≥ 3, <5, or <10 ng/mL). In the PR7 trial, this PSA threshold was set at >3.0 ng/mL after primary therapy [14]. More recently, a PSA ≥ 2 ng/mL after RT or ≥1 ng/mL after RP were used to initiate systemic therapy in the EMBARK study [10].

Several factors contribute to the heterogeneity observed across the studies evaluated in this review. Variability arises from differences in treatment populations, such as patient demographics, disease characteristics, and prior therapies. The thresholds for initiating ADT based on prostate-specific antigen PSA levels also vary significantly, with some studies adopting lower thresholds and others allowing for higher PSA levels before treatment. Furthermore, the type and duration of ADT employed differ across studies, ranging from intermittent to continuous regimens, and sometimes incorporating ARPIs as part of the therapeutic strategy. These inconsistencies, coupled with diverse definitions of early versus delayed treatment, complicate the interpretation and comparison of findings.

Given the heterogeneity between BCR study populations, the reviewed studies do not allow us to clearly define the optimal timing or exact PSA threshold for ADT initiation. However, studies clearly show that prognostic factors play a role in determining management, which, in part, endorses the low- and high-risk scheme used in the EAU guidelines. However, more recently, the EMBARK trial has shown that early systemic treatment with ARPI + ADT can improve cancer outcomes for men with high-risk BCR after local therapy. Indeed, EMBARK clearly demonstrates that combining an ARPI with ADT or an ARPI alone may be superior to ADT alone in this setting.

However, in the absence of consistent evidence indicating a significant survival advantage for patients with low-risk BCR, it is possible that initiating early ADT does not offer benefits to this subgroup and could instead pose potential harm secondary to ADT’s unsavory toxicity profile. The primary aim of early ADT administration is to defer disease progression, extend overall survival, and enhance patients’ QOL. Considerations such as the onset of hot flashes, fatigue, a diminished libido, sarcopenia, osteoporosis, and the potential elevation in cardio-metabolic consequences including diabetes and coronary diseases are pivotal when contemplating the initiation of ADT. A study conducted at the Memorial Sloan-Kettering Cancer Center revealed elevated levels of fatigue, emotional distress, and sexual dysfunction, and a diminished overall QOL among asymptomatic men with locally advanced PCa or PSA-only relapse who underwent early ADT compared to those who received delayed therapy [5].

At present, several guidelines address the management of men experiencing PSA relapse, and these are shown in Table 6. The current American Society of Clinical Oncology (ASCO) guideline recommends deferring ADT for patients wishing to avoid or delay potential side effects and for asymptomatic patients. Meanwhile, early intermittent ADT is suggested for patients with higher-risk BCR, including those with a PSADT of less than 10–12 months, a GS ≥ 8, or an interval to BCR ≤ 18 months [41]. Similarly, the National Comprehensive Cancer Network (NCCN) guideline proposes that patients with elevated PSA levels or a short PSADT and a long-life expectancy may benefit from initiating ADT earlier [42]. 

In contrast, the European Society for Medical Oncology (ESMO) guidelines do not recommend initiating ADT uniformly [43]. The European Association of Urology (EAU) guidelines stratify BCR patients into low- and high-risk groups, suggesting that early ADT should be reserved for those at high risk of disease progression, characterized by a short PSADT at relapse (≤12 months), a high initial International Society of Urological Pathology (ISUP) grade, and a long-life expectancy. ADT should not be offered to non-metastatic patients with a PSADT greater than 12 months [44]. On a different note, the National Institute for Health and Care Excellence (NICE) guidelines state that a rising PSA alone does not necessarily warrant an immediate change in treatment, and routine ADT should not be initiated for patients with BCR unless they exhibit symptomatic local disease progression, confirmed metastases, or a PSADT of less than 3 months [45].

**Table 6:** Summary of available guidelines.

Guidelines for Biochemical Recurrence without Metastatic Disease	
ASCO [41]	Early intermittent ADT can be offered to patients with higher-risk BCR, including those with a PSADT of less than 10–12 months, a GS ≥ 8, or a time to BCR interval ≤18 months. Deferred ADT is often the preferred option for patients who wish to avoid or delay the potential side effects associated with ADT. It is crucial to carefully consider offering deferred ADT only to asymptomatic patients.
NCCN [42]	The timing of ADT initiation should be personalized based on factors such as PSA velocity, patient anxiety, and potential side effects. In particular, patients with a shorter PSADT or rapid PSA velocity, coupled with a long-life expectancy, may benefit from early initiation of ADT. Addition of enzalutamide could be useful in certain circumstances. On the other hand, patients with longer PSADTs, especially those who are older, may be better suited for an observation approach, monitoring their condition without immediate ADT intervention.
AUA/SUO [46]	Clinicians should offer observation or clinical trial enrollment. Clinicians should not routinely initiate ADT. Clinicians may offer intermittent ADT in lieu of continuous ADT, if ADT is initiated in the absence of metastatic disease Clinicians should utilize PSMA PET imaging preferentially, as an alternative to conventional imaging due to its greater sensitivity, or in the setting of negative conventional imaging. (Expert Opinion)
EAU-EANM-ESTRO-ESUR-ISUP-SIOG [44]	Early ADT should be reserved for those at the highest risk of disease progression defined mainly by a short PSADT at relapse (≤12 months) or a high initial ISUP grade (4–5) and a long-life expectancy. Offer enzalutamide with or without ADT to patients with M0 disease with high-risk BCR, defined as a PSA doubling time of 9months andPSA 2 ng/mL above the nadir after RT or 1 ng/mL after RP with or without postoperative RT. ADT initiation in non-metastatic patients with a PSADT >12 months is not recommended.
ESMO [43]	Early ADT alone is not recommended for men with biochemical relapse unless they have a rapid PSA doubling time, symptomatic local disease or proven metastases.
NICE (National Institute for Health & Care Excellence) UK [45]	BCR alone should not warrant an immediate change in treatment. PSADT should be estimated if BCR occurs and should be determined from at least three measurements over a 6-month period. Enrollment in ongoing clinical trials should be considered. Routine administration of ADT should not be offered to individuals with PCa solely based on BCR, unless they meet certain criteria: Symptomatic local disease progression Presence of proven metastases PSADT of less than three months

As new data continue to emerge, the implementation of findings from studies like EMBARK underscores the need for guideline updates to enlighten clinicians and better optimize patient management in this setting. Consequently, we advocate for the development of a novel treatment algorithm. While considering insights from previous guidelines, our proposed algorithm summarizes findings from this review and integrates hormonal treatment intensification that is specifically tailored to address the needs of the high-risk biochemically recurrent patient population.

Patient selection remains important. Foremost, these guidelines should apply to patients who present with BCR, defined as patients with PSA-only progression without visible metastases on conventional imaging that are post-RT or -RP or that are not eligible for salvage therapy such as salvage RT and RP. 

As a preamble, genomic markers may also, in the future, help to better guide treatment in this setting. While PSMA PET scans are being integrated into patient care and disease staging more often, further trials are needed to define their role in this setting and determine the best course of treatment for PSMA-avid disease in the context of PSA-only disease and negative conventional imaging. Indeed, a recent study looked at the role of PSMA-PET in a patient population representative of EMBARK. Indeed, a post-hoc analysis was performed on 146 patients from six prospective studies who underwent a PSMA-PET scan while meeting the inclusion/exclusion criteria of EMBARK. Interestingly enough, PSMA PET scans yielded positive results in 83% of cases, while identifying metastatic disease in 40% of patients, with nearly 20% presenting with five or more lesions [47]. In this context, one may theorize that some of the benefits of treatment intensification seen in the EMBARK study may derive from giving ARATs to oligometastatic and plurimetastatic disease, which, in the absence of a PSMA PET, may have gone undetected and may have been sub-optimally treated with ADT + placebo. This becomes more important when we know that the early addition of an ARPI to ADT in the metastatic setting leads to a true survival gain, not just a lead time bias, which could be recuperated by adding ARPI later. However, although EMBARK and PRESTO did not consider the addition of a PSMA PET, the currently accruing phase III RCT ARASTEP, which is comparing placebo to Darolutamide in high-risk BCR PCa patients, may help shed light on its use in this setting [12]. 

In the absence of definite evidence, we suggest that, if a patient presents with plurimetastatic disease on PSMA PET but negative disease on conventional imaging and otherwise with a PSA-only recurrence, the patient may be suitable for these guidelines and would benefit substantially from ADT + enzalutamide. If a patient presents with PSMA-avid oligometastatic disease, which may correspond to non-specific but targetable lesions on conventional imaging, that is amenable to targeted RT, given the lack of data in this setting, we would still but tentatively recommend SBRT while treating with ADT + enzalutamide as per EMBARK.

Patients with BCR who are ineligible for salvage treatments should first be stratified into low- and high-risk groups. High-risk classification should be based on the following criteria: PSADT ≤ 12 months, PSA levels of ≥1 ng/mL post-RP or ≥2 ng/mL post-RT, a time to BCR of ≤18 months (as per ASCO guidelines), a Gleason score (GS) ≥ 8 and a long life expectancy (EAU/ESTRO).

Patients who do not meet these high-risk criteria should be stratified into the low-risk group. These patients should not be started on early ADT. Instead, they should continue with regular follow-ups by their clinician under active surveillance. For patients in the low-risk group, intermittent ADT may be considered if their PSA levels rise to ≥5 ng/mL. However, if the PSADT shortens or metastases develop, subsequent treatment should be initiated.

We recommend, however, that a personalized approach be taken with every patient. Shared decision-making should be practiced when deciding on an approach. The type and timing of treatment should also take into account the PSA velocity, patient anxiety, and potential side effects. Older individuals with a longer PSADT or who are unfit to receive ADT or an ARPI may be more suitable for observation (NCCN).

Clinicians should not routinely initiate ADT in low-risk patients. Observation or enrollment in a clinical trial would be the preferred option for this patient group (AUA/ASTRO/SUO/EAU/ESTRO). BCR alone should not warrant an immediate change in treatment. The PSADT should be estimated from at least three measurements over a 6-month period. However, the integration of PSMA PET for the early detection of metastases may be considered. The treatment of actionable oligo metastases may be an option. 

For patients meeting high-risk criteria, the preferred treatment option would be enzalutamide and ADT. As per EMBARK, treatment should be given for 37 weeks or roughly 9 months. If the PSA reaches a value < 0.2 ng/mL, patients can be stopped and observed. If the PSA increases to 2.0 ng/mL post-RP or ≥5.0 ng/mL post-RT without RP, combined treatment should be restarted. If the PSA does not reach a value < 0.2 ng/mL, treatment with ADT and enzalutamide should be continued and re-assessed at disease progression. For patients who are ineligible or do not want to undergo combined therapy, if eligible, they can receive enzalutamide in a similar regimen. If patients do not want to receive an ARPI, then, if eligible, they should undergo intermittent ADT. Early ADT initiation can also be considered in patients with a PSADT < 12 months, after a detailed discussion with the patients of the risk and benefits, but should not be considered for those with a PSADT >12 months (EAU/ESTRO).

**Figure 3:**
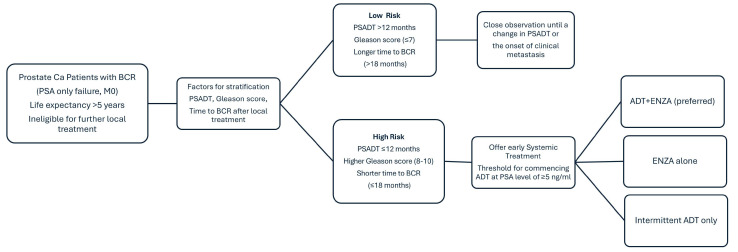
Proposed guideline for biochemical recurrent prostate cancer patients. (PSA: Prostate specific Antigen, BCR: Biochemical recurrence, PSADT: PSA doubling time, ENZA: enzalutamide, ADT: Androgen deprivation therapy).

The objective of this review was to better delineate the treatment landscape of BCR-only PCa. By an extensive review of the literature and integration of novel treatment strategies, we believe that we have produced comprehensive and practical guidelines for patients with PSA-only recurrence, as shown in Figure 3. Trials like EMBARK were sorely lacking in representation of the non-metastatic BCR state, where, until recently, the standard of care revolved around intermittent ADT alone. The combination of ADT + enzalutamide has now transformed treatment for high-risk BCR patients. A weakness of this review is that long-term data are lacking, and other relevant trials have not matured or even accrued yet. In addition, more studies validating the use and integration of PSMA PET are needed to better define its use in this setting [47]. In the future, stratification may encompass PSMA PET staging and the use of genomic markers to better allocate patients requiring treatment from those who may safely avoid its associated toxicities [48]. Nonetheless, one may perceive this as the dawn of an era of utilizing ARPIs in the high-risk BCR landscape [9].

## 5. Conclusions

This review emphasizes the critical need for personalized decision-making when considering the use of ADT in patients experiencing BCR and PSA relapse as the sole indicator of cancer progression. After reviewing the guidelines, we have formulated our own recommendations. Patients with unfavorable prognostic factors, such as a short PSADT (<12 months), a higher GS (8–10), and a shorter time to BCR (<18 months), with a PSA ≥ 1 ng/mL post-RP or a PSA ≥ 2 ng/mL post-RT may benefit from the early initiation of ADT. Conversely, those with favorable prognostic factors, including a longer PSADT, lower GS, and longer time to BCR, may be suitable candidates for close observation until there is a change in their PSADT or the development of clinical metastasis. For patients in this low-risk group, intermittent ADT could also be an option if the PSA rises to ≥5 ng/mL. Combining ADT with ARPIs such as enzalutamide is recommended for patients stratified into the high-risk category to more effectively block the androgen receptor pathway. However, for patients who cannot tolerate the combination therapy, or who wish to avoid certain adverse effects, enzalutamide alone or intermittent ADT remain viable and acceptable treatment options. This tailored approach aims to optimize therapeutic outcomes while minimizing potential side effects, highlighting the importance of individualized care in the management of BCR in PCa. Clinicians are advised to prioritize PSMA PET imaging where it is accessible. This approach serves as an alternative to conventional imaging due to its enhanced sensitivity. Additionally, PSMA PET imaging can be particularly valuable in situations where conventional imaging results are negative. In the management of PSMA-avid oligometastatic disease, where lesions are identifiable on conventional imaging, we suggest considering the integration of SBRT alongside ADT with enzalutamide. While data may be limited, the tentative application of SBRT in this context aims to target specific lesions, potentially delaying the progression of the disease and maintaining the patient’s quality of life. 

## Figures and Tables

**Table 1:** Summarizes the keywords and search results used in each database.

Database	Search Results	Keywords
PubMed	182	Prostate neoplasm, androgen deprivation, androgen receptor antagonists, biochemical recurrence, salvage therapy, time to treatment, delayed treatment, Prostate-Specific Antigen, treatment failure
Embase	88	
Cochrane	25	

**Table 2:** Summary of retrospective studies evaluating the role of ADT in BCR post-surgery and RT.

Primary Treatment: Surgery +/−RT									
Author	Study Type	Year of Publication	No. of Patients	Intervention	Outcomes Measured	Follow-Up Length	Previous Local Treatment	Results	**Conclusion**
Roderick C.N. van den Bergh et al. [7]	Systematic Review	2015	11,606	2 RCTs 8 NRS 17 case series	OS, CSS, FDM, QoL, time to CRPC	Different range in selected studies	RP +/− Adj EBRT Post Definitive EBRT	Conflicting results. Factors predictive for poor outcomes (CRPC, DM, CSS, OS): Short PSADT, High GS, High PSA, Increased age, and comorbidities.	Early ADT should be reserved for patients with highest risk of disease progression, (short PSADT < 6–12 months, high initial GS (>7), and a long-life expectancy.
Duchesne et al. (TOAD Trial) [13]	P-III RCT	2016	293	Start of ADT < 8 weeks ADT ≥ 24 months	OS	Median follow-up 5 years (IQR 3·3–6·2)	(Group I) RT 63.2% RP +/− EBRT 36.8%	5-year OS was 86·4% (95% CI 78·5–91·5) in the delayed therapy arm vs. 91·2% (84·2–95·2) in the immediate therapy arm (*p* = 0·047).	Immediate receipt of ADT significantly improved OS compared with delayed intervention.
Crook et al. [14]	P-III RCT	2012	1386	Start of ADT at PSA > 3 ng/mL (continuous vs. intermittent)	OS, QoL, time to CRPC	Median follow-up 6.9 years (range, 2.8 to 11.2)	RP+ Salvage RT (11.4%) Only RT (88.5%)	Median OS was 8.8 years in iADT group and 9.1 years in continuous-therapy group.	iADT was noninferior to continuous therapy with respect to OS. Some QOL factors improved with intermittent therapy.
Ahn et al. [15]	Retrospective	2020	69/923	PSA level at ADT initiation <2 vs. >2 ng/mL	Progression to CRPC-survival, CSS	Median follow-up 81 months (IQR 54.2–115.7)	RP + Adjuvant/salvage EBRT	On UVA, GS ≥ 8 (*p* = 0.024), PSA at ADT initiation ≥ 2 ng/mL (*p* = 0.015), were significantly associated with an increased risk of CSS. On MVA, PSA at ADT initiation ≥ 2 ng/mL was an independent predictor of CSS.	Delaying ADT for PSA beyond 2 ng/mL was associated with an increased risk of progression to CRPC and CSM.
X. Garcia-Albeniz et al. [16]	Retrospective	2015	2096	Start of ADT < 3 months ADT > 24 months	ACM	Mean follow-up 54.0 months (SD 38.6)	RP +/− RT (68.6%) EBRT and/or Brachytherapy 31.4%	Similar 5-year survival difference between groups (adjusted mortality HR for immediate vs. deferred ADT: 0.91 (95% CI, 0.52 to 1.60).	Immediate ADT initiation within 3 months had similar survival to deferred ADT initiation.
Alez Z. Fu et al. [17]	Retrospective	2017	5804	Start of ADT < 12 months ADT > 12 months	PCSM, ACM	Median follow-up 8 years (range 2–15)	RP 46.1% RT 53.9%	Early ADT was associated with neither ACM nor PCSM within the RP or the RT cohort. ADT was statistically significantly associated with decreased risk of ACM and PCSM within prostatectomy and the RT cohort with PSADT < 9 months.	No association of ADT with ACM or CSM. Men with rapidly progressing disease (PSA doubling time < 9 months) may benefit from early ADT.
De la Taille et al. [18]	Case series	2002	74	IADT Mean threshold PSA 10 ng/mL	PFS, OS, FDM	Mean follow-up 43.8 months	RP + RT (21.6%) RP (40.5%) RT (37.8%)	Overall, 5-year bPFS rate was 54.6%. On MVA, factors predictive of biochemical progression were age < 70 years (*p* = 0.05), GS ≥8 (*p* = 0.038) and the presence of lymph node metastases (*p* = 0.05).	IADT is a treatment option for patients with BCR after local treatment. Candidates for iADT must be >70 years, with localized adenocarcinoma and a GS ≤ 7.
Keizman et al. [19]	Retrospective	2011	96	IADT PSA threshold for initiation of IAD 10–20 ng/mL	CPFS	Median follow-up 65 months (22–183)	RP 80% RT 19%	Factors associated with disease progression were pre-treatment PSADT (≥6 vs. <6), first off treatment interval PSADT (≥3 vs. <3), and PSA nadir during the first treatment interval (<0.1 vs. ≥0.1).	PSADT before treatment and during the first off treatment interval is associated with disease progression.

RT: Radiotherapy, RCT: randomized control trial, ADT: androgen deprivation therapy, GS: Gleason score, FDM: freedom from distant metastasis, OS: overall survival, CSS: cancer-specific survival, CRPC: castrate-resistant prostate cancer, PCSM: prostate cancer-Specific mortality, ACM: all-cause mortality, CSM: cause-specific mortality, LF: local failure IAD: intermittent ADT, CPFS: clinical progression-free survival, bPFS: biochemical progression-free survival, IADT: intermittent androgen deprivation therapy, IQR: interquartile range, MVA: multivariate analysis, UVA: univariate analysis, QOL: quality of life. PCa: Prostate Cancer, PSADT: Prostate specific antigen doubling time, BCR: biochemical recurrence.

**Table 3:** Summary of retrospective studies evaluating timing of ADT in BCR post-RT.

Primary Treatment: RT only									
Author	Study Type	Year of Publication	No. of Patients	Intervention and Outcomes Measured	Outcomes Measured	Follow-Up Length	Previous Local Treatment	Results	**Conclusion**
Brandon A. Mahal et al. [20]	Retrospective	2018	108	Start of ADT PSA < 10 ng/mL PSA > 10 ng/mL	PCSM	Median follow-up 5.68 years (IQR range, 3.05–9.56)	EBRT 100%	Amongst men with a long PSADT (≥6 months), initiating ADT later PSA > 12 ng/mL, versus earlier was associated with an increased risk of PCSM (AHR 8.84, 95% CI 1.99–39.27; *p* = 0.004); whereas this was not true for men with a short (<6 month) PSADT (*p* = 0.79).	Early initiation of ADT at lower absolute PSA levels for men with a PSADT of ≥6 months or more may reduce the risk of PCSM.
Sagalovich et al. [21]	Retrospective	2016	109	Start of ADT < 3 months ADT > 3 months	PCSM ACM	Median follow-up 11.4 years [IQR]: 8.0–14.3	Brachytherapy +/− EBRT 100%	No difference in PCSM for patients receiving delayed vs. immediate ADT.	Shorter PSADT and time to BCR are significantly associated with adverse outcomes, and these patients should be considered for immediate ADT
Pinover et al. [22]	Retrospective	2003	248	Use of ADT in patients with PSADT < 12 months vs. PSADT > 12 months	FDM CSS, OS	Median follow-up 46 months (Range 6–95)	EBRT 100%	Significant improvement in 5-year FDM rate in men with PSADT < 12 months who started ADT compared to observation. No improvement in FDM with use of ADT in patient with PSADT > 12 months.	The results validate the use of PSADT as an indicator of patients who may be observed expectantly or treated with ADT for PSA failure after RT.
Kestin et al. [23]	Retrospective	2004	1201	Early ADT at BCR >/= 3 ng/mL above nadir Delayed ADT at clinical failure	DM. CSS, OS	Median follow-up 6.6 years (range, 0.1–14.8 years)	EBRT 100%	5-year clinical failure was 60% for patients who experienced a PSA rise to ≥3 ng/mL above nadir. For these patients, early ADT was associated with decreased 5-year DM (13% vs. 44%), CSS (9% vs. 24%), and death due to any cause (32% vs. 48%).	PSA elevation to ≥2 or ≥3 ng/mL above nadir seems optimal in establishing clinically significant BCR and the timing of ADT intervention.
Klayton et al. [24]	Retrospective	2011	432	Use of ADT in patients with PSADT <6 months’ vs. PSADT > 6 months	FDM, CSS, OS	Median follow-up 95 months (6–207)	EBRT 100%	Independent predictors of FDM were PSADT (*p* < 0.0001), GS (*p* = 0.011), and the use of initial ADT (*p* = 0.005). There was no benefit to ADT in 7 years CSS for patients with PSADT > 6 months.	Immediate use of ADT in patients with PSADT < 6 months is significantly associated with improved CSS, although the benefit is less apparent in patients with longer PSADT
Kim et al. [25]	Retrospective	2013	108	ADT started at PSA 10 ng/ml	ACM	Median follow-up 10.3 years (IQR = 8.0–11.9)	EBRT 100%	Increasing PSADT was associated with a significant increase in the risk of death (adjusted HR, 1.21; 95% CI 1.02–1.45; *p* = 0.03).	Despite unfavorable PSA kinetics at recurrence, unhealthy men may not benefit from ADT; for healthy men with unfavorable PSA kinetics at recurrence, PC death rates are high despite ADT.
Kim-Sing et al. [26]	Retrospective	2004	465	Effect of PSADT, time to BCR on timing of ADT	CSS	Median follow-up 71 months	EBRT 100%	The 5-year CSS was 19% where the PSADT was <3 months, 84% where 3–6 months, 93% where 6–12 months, and 98% where >12 months (*p* < 0.0001).	Men with PSADT > 1 year had excellent 5-year CSS (98%). Rapid PSADT is associated with increased mortality on MVA.
Shipley et al. [27]	Retrospective	2006	143/247 Subset of patients	Impact of salvage ADT timing in patients treated with RTOG-8610 protocol	OS, CSS	Median follow-up 9.0 years	EBRT 100%	A statistically significant increase in CSS was observed when patients with PSA < 20 were compared with those with PSA > 20 at the time salvage ADT was started.	This protocol could not evaluate the effect of post-treatment PSADT on outcomes, and could not significantly demonstrate that early salvage ADT in patients with long post-treatment PSADT is necessary for longer survival.
Martin et al. [28]	Retrospective	2014	53/108	Observation only after BCR	OS, CSS	Median follow-up 4.0 years (IQR: 2.0–12.5)	EBRT 100%	Both increasing age at PSA failure (HR: 1.14; 95% CI: 1.03–1.25; *p* = 0.008) and the presence of moderate to severe comorbidity (HR: 12.5; 95% CI: 3.81–41.0; *p* < 0.001) were significantly associated with an increased risk of death.	Men >76 with significant comorbidity and a PSADT >2 years appear to be good candidates for observation without ADT intervention.
Tenenholz et al. [29]	Retrospective	2007	124	ADT after BCR or after clinical metastasis	OS, DSS	Median follow-up 6.2 years	EBRT 100%	The 5-year OS was 78% when ADT was initiated at PSADT ≤ 7 months and 93% when initiated at PSADT > 7 months. Survival for patients started on ADT with doubling time < 5 months was similar to that of patients with clinical metastases.	Survival benefit justifies the use of ADT in patients whose PSADT approaches 7 months.
Souhami et al. [30]	Retrospective Secondary analysis of RTOG 85-31	2010	464	Start of Early ADT PSA < 10 ng/mL or Late ADT at PSA ≥ 10 ng/mL	OS, CSM, LF	Median follow-up 8.5 years (range, 1.4–15.1)	EBRT 100%	OS was significantly longer in the early salvage ADT group (hazard ratio, 1.5; *p* = 0.01). However, there were no statistically significant differences in LF or CSM between the groups.	The early introduction of salvage ADT resulted in improved OS but not improved CSM and LF.
Mydin et al. [31]	Retrospective secondary analysis of patients with BCR Irish Clinical Oncology Research Group 97-01	2012	102	Start of Early ADT PSA < 10 ng/mL or Late ADT at PSA ≥ 10 ng/mL	OS	Median follow-up 8.5 years	EBRT 100%	On MVA, timing of salvage ADT, time from end of RT to BCR, and PSA nadir on salvage ADT were significant predictors of survival.	Early salvage ADT based on PSA ≤ 10 ng/mL and absent distant metastases improved survival in patients with PCa after failure of initial treatment.

RT: Radiotherapy, RCT: randomized control trial, ADT: androgen deprivation therapy, GS: Gleason score, FDM: freedom from distant metastasis, OS: overall survival, CSS: cancer-specific survival, CRPC: castrate-resistant prostate cancer, PCSM: prostate cancer-specific mortality, ACM: all-cause mortality, CSM: cause-specific mortality, LF: local failure IAD: intermittent ADT, CPFS: clinical progression-free survival, bPFS: biochemical progression-free survival, IADT: intermittent androgen deprivation therapy, IQR: interquartile range, MVA: multivariate analysis, UVA: univariate analysis, QOL: quality of life.PCa: Prostate Cancer, PSADT: Prostate specific antigen doubling time, BCR: biochemical recurrence

**Table 4:** Summary of retrospective studies evaluating timing of ADT in BCR post-surgery.

Primary Treatment Surgery only									
Author	Study Type	Year of Publication	No. of Patients	Intervention	Outcomes Measured	Follow-Up Length	Previous Local Treatment	Results	Conclusion
Moul et al. [32]	Retrospective	2004	1352	Early ADT at PSA 5 or <10 ng/mL No ADT or ADT started at PSA > 10	Development of clinical metastasis	Median follow-up 3.7 years (range 0.1 to 13.0)	Surgery	Early ADT was associated with delayed clinical metastasis in patients with GS 7 or PSADT ≤12 months or less (HR = 2.12, *p* = 0.01).	Early ADT administered for BCR after prior RP was an independent predictor of delayed clinical metastases only for high-risk cases.
Siddiqui et al. [33]	Retrospective	2008	343	Adjuvant ADT or ADT at BCR 0.4 vs. No ADT until 6 months at least	PFS, CSS	Median follow-up 10 years	Surgery	Men who started ADT at a postoperative PSA of 0.4 or greater, 1.0, or 2.0 did not have improved systemic PFS or CSS.	Early ADT versus late ADT (at least 6 months after BCR) results in a worse 10 yr CSS outcome in patients with a PSA > 2 ng/mL
Antonarakis et al. [34]	Retrospective	2010	346/6401	Patients with BCR & no additional therapy until the time of radiographic metastatic disease.	MFS ACM	Median follow-up 8.6 years	Surgery	GS, pathological stage, time to PSA relapse and PSADT, age were predictive of OS and/or MFS in univariate analysis, although only PSADT (≥9 vs. 3–8.9 vs. <3 months) remained independently predictive of these outcomes in multivariate analysis (*p* < 0.001).	Dataset shows that OS and MFS can be extensive for BCR, even in the absence of further therapy before metastasis.
Marshall et al. [35]	Retrospective	2021	806	Patients with BCR & PSADT of <10 months not started on ADT until clinical metastasis	MFS, OS	Median Follow-up 9.0 years (IQR 5.0, 14.0)	Surgery	Median MFS of men with a PSADT <6 and 10 months who delay ADT until metastasis is 144 months (95% CI 48–not reached) and 192 months (95% CI 72–not reached), respectively.	Men who defer ADT until metastasis have OS and MFS that is quite long and the early initiation of continuous ADT for BCR may not meaningfully improve OS.

ADT: androgen deprivation therapy, GS: Gleason score, RP: Radical Prostatectomy, OS: overall survival, CSS: cancer-specific survival, CRPC: castrate-resistant prostate cancer, PCSM: prostate cancer-Specific mortality, ACM: all-cause mortality, CSM: cause-specific mortality, PFS: progression-free survival, IADT: intermittent androgen deprivation therapy, IQR: interquartile range, PCa: Prostate Cancer, PSADT: Prostate specific antigen doubling time, MFS, BCR: biochemical recurrence, MFS: Metastasis free survival, BCR: biochemical recurrence.

**Table 5:** Summary of RCT evaluating the role of ADT + ARPI post-surgery and RT.

Primary Treatment: Mixed (Surgery +/− RT)									
Author	Study Type	Year of Publication	No. of Patients	Intervention	Outcomes Measured	Follow-Up Length	Previous Local Treatment	Results	**Conclusion**
Freedland SJ et al. [10]	P-III RCT	2023	1068	PSADT ≤ 9 months ------------- ADT + Enzalutamide ---------- ADT alone --------- Enzalutamide alone	MFS, OS and other	Median follow-up 60.7 months	RP = 25% RT = 24.2% RP + RT = 50.4% ------------- RP = 20.9% RT = 29.1% ------------- RP + RT = 50% RP = 27.9% ------------ RT = 25.4% RP + RT = 46.8%	At 5 years, MFS was 87.3% (95% [CI], 83.0 to 90.6) in the combination group, 71.4% (95% CI, 65.7 to 76.3) in the leuprolide-alone group, and 80.0% (95% CI, 75.0 to 84.1) in the monotherapy group. With respect to MFS, enzalutamide plus leuprolide was superior to leuprolide alone (HR for metastasis or death, 0.42; 95% CI, 0.30 to 0.61; *p* < 0.001); No new safety signals were observed, with no substantial differences in quality-of-life measures between groups.	Patients with high-risk BCR, enzalutamide plus leuprolide was superior to leuprolide alone with respect to MFS; enzalutamide monotherapy was also superior to leuprolide alone. The safety profile of enzalutamide was consistent with that shown in previous clinical studies, with no apparent detrimental effect on quality of life.

RCT: randomized control trial, ADT: androgen deprivation therapy, OS: overall survival, LF: local failure. MFS: metastasis free survival, ARPI: androgen receptor pathway inhibitors, BCR: biochemical recurrence, RP: Radical prostatectomy, RT: Radiotherapy, PSADT: prostate specific antigen doubling time.

## Data Availability

No new data were created or analyzed in this study. Data sharing is not applicable to this article.
